# N‐(1H‐Indazolyl) Aryl Sulfonamide Hybrids as Potential Dihydropteroate Synthase Inhibitors: In Vitro Antibacterial Activity Against *Escherichia coli* and *Staphylococcus aureus* and Computational Modeling

**DOI:** 10.1002/cbdv.71656

**Published:** 2026-09-04

**Authors:** Romaisaa Boudza, Ferdaous Elandaloussi, Khalid Boujdi, Jaume Segura‐Garcia, Nabil El Brahmi, Saïd El Kazzouli, Ismaïl Moukadiri, Sergi Maicas, Salim Bounou

**Affiliations:** ^1^ Euromed University of Fes, UEMF Morocco; ^2^ Department of Microbiology and Ecology. Faculty of Biology Universitat De València Burjassot Spain; ^3^ Faculty of Sciences and Techniques of Tangier Tangier Morocco; ^4^ Department of Computer Science, School of Engineering Universitat De València Burjassot Spain

**Keywords:** ADME, antimicrobial resistance, dihydropteroate synthase, drug design, indazole, molecular docking, sulfonamide

## Abstract

Antimicrobial resistance (AMR) has severely compromised the clinical utility of classical sulfonamide antibiotics, primarily owing to the emergence of resistance‐associated dihydropteroate synthase variants. In this study, sixteen N‐(1H‐indazolyl) aryl sulfonamide hybrids were evaluated as potential DHPS inhibitors against *Escherichia coli* and *Staphylococcus aureus* using disk diffusion, broth microdilution (MIC_80_), and minimum bactericidal concentration (MBC) assays. Active compounds were characterized by in silico ADME and toxicity profiling, with molecular docking performed against wild‐type DHPS and the sulfonamide‐resistant Sul1 variant. Antibacterial activity was species‐dependent: compound 4 showed the strongest activity against *E. coli* (MIC = 32 µg/mL), while compounds 11 and 16 were most potent against *S. aureus* (MIC = 32 µg/mL), outperforming sulfathiazole and sulfisoxazole. All hybrids showed superior docking affinities to sulfamethoxazole (SMX), with compound 11 showing the highest affinity for wild‐type DHPS (−7.79 kcal/mol); compound 4 retained favorable binding against Sul1 (−6.83 kcal/mol), suggesting potential resistance resilience. Structure–activity analysis showed antibacterial efficacy is governed by aryl sulfonyl electronic effects, steric balance at the C7 indazole position, and physicochemical properties affecting Gram‐negative membrane permeability. These findings support indazole–sulfonamide hybrids as promising scaffolds that may retain predicted binding complementarity toward the Sul1‐resistant DHPS variant, warranting validation against resistant isolates and purified DHPS enzymes.

## Graphical abstract

Sixteen indazole–sulfonamide hybrids exhibit species‐dependent antibacterial activity agains*t E. coli and S. aureus*, with the lead compound displaying an eightfold lower MIC than sulfathiazole against *E. coli*. Molecular docking against wild‐type and Sul1‐resistant dihydropteroate synthase identifies a candidate retaining binding affinity toward the resistant variant. Structure‐activity and ADMET analyses position this scaffold as a promising antibacterial lead.

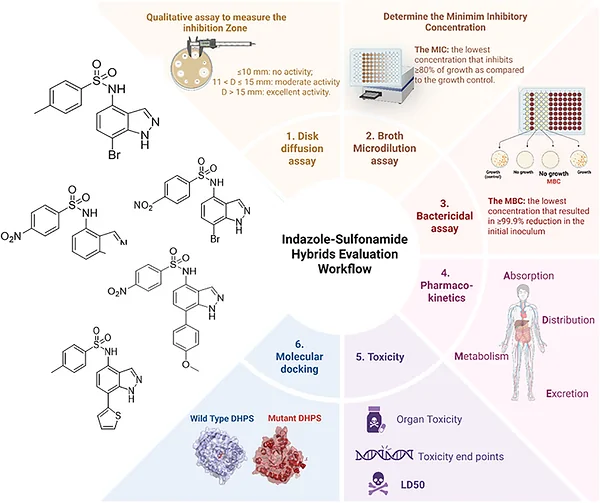

## 1 Introduction

The global burden of antimicrobial resistance (AMR) has emerged as one of the leading public health threats of the 21^st^ century, causing approximately five million deaths globally in 2019. Projections suggest that, by 2050, deaths attributable to AMR could reach 10 million annually, surpassing the global mortality burden of cancer [1]. Antibiotics are not only used to treat common, nonlife‐threatening infections; they are also critical as prophylactic agents for the prevention and treatment of opportunistic infections in patients undergoing intensive chemotherapy, organ transplantation, or living with advanced HIV [2, 3]. Without effective antibiotics, simple bacterial infections in these vulnerable patients could rapidly escalate to life‐threatening sepsis [4, 5].

One cornerstone therapy is the trimethoprim/sulfamethoxazole combination (TMP‐SMX), which is widely used to prevent opportunistic infections in immunosuppressed patients [6]. Introduced in the 1980s, TMP‐SMX has remained a first‐line prophylactic antibiotic, notably in the prevention of *Pneumocystis jirovecii* pneumonia and other infections in patients with profound neutropenia or HIV/AIDS [7, 8, 9]. Sulfamethoxazole (SMX), the sulfonamide component of this combination, carries a particularly rich legacy: it belongs to the sulfonamide class, which represents the first generation of synthetic antibiotics used in humans [10]. These compounds act as competitive inhibitors of *p*‐aminobenzoic acid (PABA) by targeting the enzyme dihydropteroate synthase (DHPS), which catalyzes the condensation of PABA and 6‐hydroxymethyl‐7,8‐dihydropterin‐pyrophosphate (DHPPP) to form dihydropteroate (DHPt) [11]. The binding of sulfonamides inhibits this reaction, leading to a “dead‐end” sulfa‐pterin product and suppressing the synthesis of folate, which is essential for the production of thymidine and purine nucleotides required for bacterial DNA synthesis and cell replication [12]. The specificity of this mechanism also explains the safety of sulfonamides in humans: human cells lack DHPS and must acquire folate from the diet, so the drug acts selectively on microorganisms without affecting host cells [13].

Co‐trimoxazole and other sulfonamide‐containing combinations remain clinically relevant for the treatment of a wide variety of bacterial and protozoal infections [14]. These include uncomplicated and complicated urinary tract infections caused by susceptible *Escherichia coli* or *Klebsiella pneumoniae*, as well as skin and soft tissue infections attributable to community‐acquired *Staphylococcus aureus* [15, 16]. In addition, sulfonamides continue to be used in topical formulations, such as silver sulfadiazine, for the prevention and treatment of burn‐associated infections [17].

However, the clinical effectiveness of sulfonamides has been progressively undermined by the emergence and dissemination of bacterial resistance [18]. The predominant resistance mechanism is the acquisition of alternative DHPS enzymes encoded by *sul* genes (*sul1*, *sul2*, and *sul3*), which are predominantly located on plasmids and integrons. These genes encode sulfonamide‐insensitive DHPS variants that retain catalytic activity in folate biosynthesis but exhibit markedly reduced affinity for sulfonamides, thereby bypassing competitive inhibition [10]. Such resistance determinants are widely distributed among clinical isolates of Gram‐negative pathogens such as *E. coli, A. baumannii*, and *K. pneumoniae* [19, 20, 21]. Additional resistance mechanisms include point mutations in the chromosomal *folP* (encoding DHPS) that alter the sulfonamide‐binding pocket [6]. Recent structural and crystallographic studies of the sulfonamide‐resistant enzyme Sul1 have provided molecular‐level insights into how subtle active‐site rearrangements preserve physiological substrate binding while sterically and electrostatically disfavoring sulfonamide interaction, thereby explaining the persistence of resistance and highlighting opportunities for resistance‐aware drug design [6, 10].

In parallel, the marked slowdown in the discovery of truly novel antimicrobial classes since the mid‐twentieth‐century “golden era” of antibiotic development has increased interest in repurposing and optimizing established drug scaffolds rather than pursuing solely *de novo* targets [22, 23]. In this context, the sulfonamide scaffold remains highly relevant owing to its chemical versatility, well‐validated mechanism of action, and amenability to structural modification [24]. Modern medicinal chemistry efforts have increasingly focused on molecular hybridization, in which the sulfonamide pharmacophore is covalently linked to a second bioactive scaffold to enhance potency, broaden the antibacterial spectrum, or overcome resistance [25]. *N*‐heterocyclic sulfonamides have emerged as a particularly promising class, given that heterocyclic moieties can introduce additional binding interactions and modulate physicochemical properties.

Among these, the indazole scaffold has attracted significant attention as a privileged heterocycle with broad biological relevance. Several studies have reported potent antibacterial activity for both standalone indazole derivatives and various sulfonamide‐containing heterocycles [26, 27, 28]. Despite the individual promise of these two scaffolds, the systematic evaluation of indazole–sulfonamide hybrids remain remarkably limited, leaving a notable gap in the current literature. Furthermore, existing research has largely focused on phenotypic efficacy, with limited integration of resistance‐aware target interrogation. Specifically, the ability of such hybrids to retain activity against sulfonamide‐resistant dihydropteroate synthase (DHPS) variants and to mechanistically link structural modifications to target engagement remains largely unexplored. In contrast to classical sulfonamide antibiotics and previously described sulfonamide‐based DHPS inhibitors, which primarily feature simple aniline or heteroaryl backbones, the present study introduces indazole‐sulfonamide hybrids as a structurally novel compound class.

To the best of our knowledge, no comprehensive study has yet investigated whether an indazole nucleus combined with a sulfonamide pharmacophore can function as a DHPS inhibitor. This hybrid scaffold is designed to address the limitations of classical sulfonamides of decreased potency and susceptibility to resistance by providing more structural diversity in terms of interactions with the enzyme binding pocket.

The advent of computational techniques, such as molecular docking and structure‐activity relationship (SAR) analysis, has substantially advanced this field by providing molecular insights into the interaction mechanisms between bioactive compounds and their molecular targets. By combining these computational methods with in vitro evaluation, this study aims to address the existing gap by assessing indazole–sulfonamide hybrids as potential antibacterial agents designed to explore potential activity in the context of sulfonamide‐resistance‐associated DHPS variants.

In the present study, we provide in vitro evidence and preliminary computational evidence suggesting that hybridization of the sulfonamide pharmacophore with an indazole scaffold represents a promising strategy for the development of potential DHPS‐targeting antibacterial agents with possible resilience against sulfonamide resistance mechanisms. Our results reveal species‐dependent antibacterial activity, with selected compounds surpassing standard sulfonamide controls and exhibiting superior binding affinities relative to SMX. Molecular docking elucidated key residue interactions within both susceptible and resistant DHPS active sites, highlighting structural features responsible for enhanced affinity and resistance resilience. Furthermore, integrated ADME and toxicity profiling support the drug‐likeness and acceptable safety profiles of the most promising derivatives. This work provides mechanistic insights into the structure–activity relationships of indazole–sulfonamide hybrids and establishes a rational foundation for the design of next‐generation DHPS‐targeted antibacterial agents.

## 2 Results and Discussion

### 2.1 Qualitative Assessment of Antimicrobial Activity

The disk diffusion assay revealed selective antibacterial activity among the tested compounds, with clear species‐dependent effects (Table 1). Against *E. coli* CECT 101, compound 4 emerged as the only highly active compound, producing inhibition zones of 17, 15, and 12 mm at 100, 50, and 25 µg per disk, respectively.

**TABLE 1:** Diameters of inhibition zones (mm) against *E. coli* CECT 101 and *S. aureus* CECT 4013.

		Inhibition zone diameter (mm)	
Molecule ID	Disc content (µg)	*E. coli* CECT 101	*S. aureus* CECT 4013
1	100, 50, 25	NE	NE
2	100	NE	**15**
	50	NE	**11**
	25	NE	NE
3	100, 50, 25	NE	NE
4	100	**17**	**11**
	50	**15**	NE
	25	**12**	NE
5	100, 50, 25	NE	NE
6	100	10	9
7	100, 50, 25	NE	NE
8	100, 50, 25	NE	NE
9	100, 50, 25	NE	NE
10	100, 50, 25	NE	NE
12	100	NE	NE
	50, 25	NE	8
13	100, 50, 25	NE	NE
14	100, 50, 25	NE	NE
15	100	NE	22
	50	NE	20
	25	NE	16
16	100	NE	12
	50	NE	13
	25	NE	11
SMX	50	31	24

Abbreviations: NE: No Effect, SMX: Sulfamethoxazole.

**FIGURE 1:**
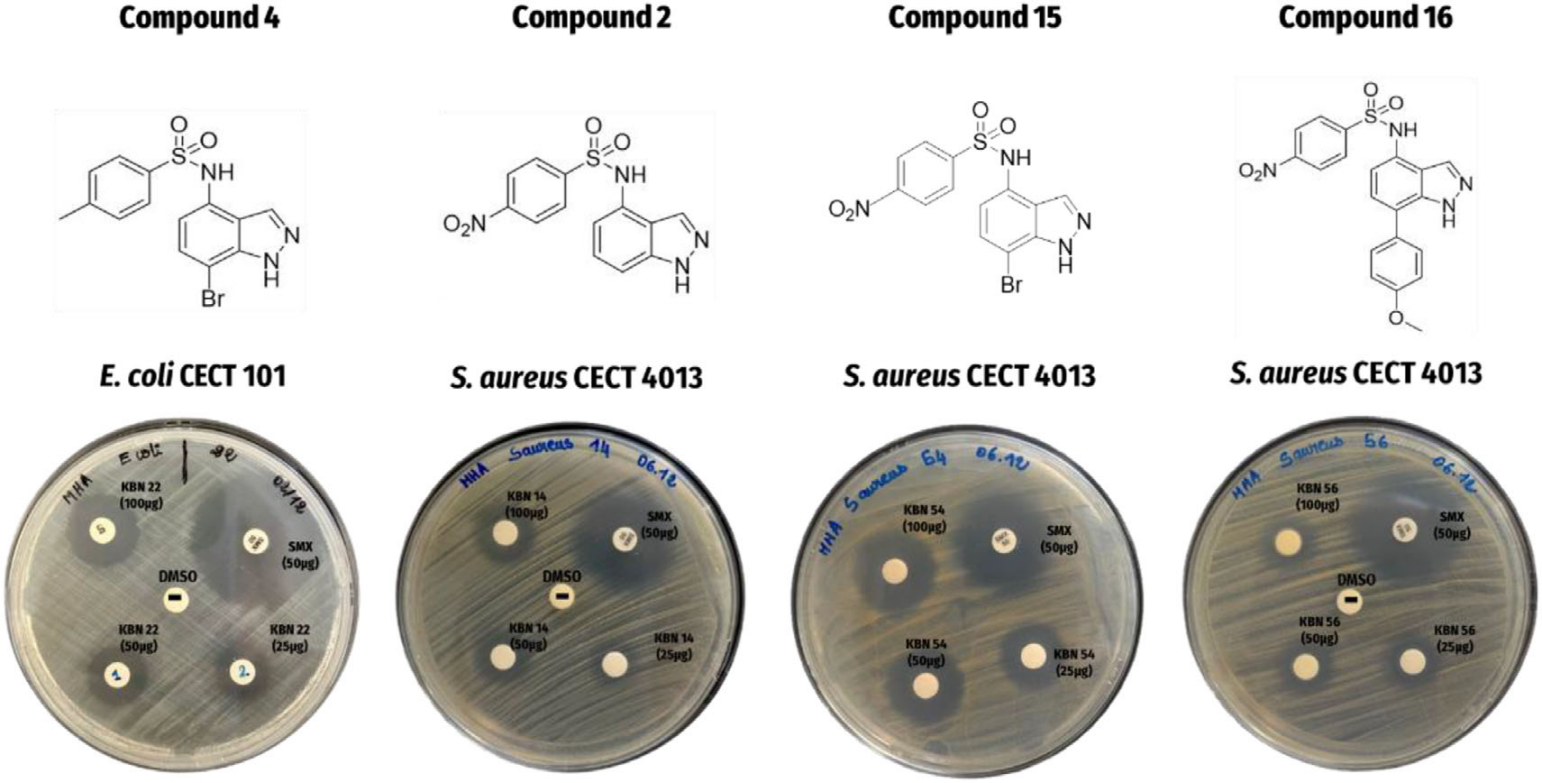
Disk diffusion assay showing inhibition zones of compounds 2, 4, 15, and 16 against *E. coli* CECT 101 and *S. aureus* CECT 4013 at 25, 50, and 100 µg per disk. Compounds 1, 3, and 5–14 showed no detectable activity (data not shown). The codes KBN14, KBN22, KBN54, and KBN56 correspond to compounds 2, 4, 15, and 16, respectively.

In contrast, *S. aureus* CECT 4013 proved more susceptible to several derivatives. Compound 15 displayed the strongest anti‐*S. aureus* activity, with inhibition zones of 22, 20, and 16 mm across the tested concentrations, approaching the efficacy of the reference antibiotic SMX (24 mm at 50 µg). Moderate activity against *S. aureus* was also observed for compound 2 (15 mm at 100 µg per disk) and compound 16 (11–13 mm across concentrations) (Figure 1).

Comparison of compounds 1, 3, and 5–14 showed no detectable inhibition zones against either bacterial strain under the experimental conditions employed.

### 2.2 Minimum Inhibitory Concentration

MIC values were determined using a twofold serial dilution method, with concentrations ranging from 8 to 1024 µg/mL, a range selected to encompass both weakly and highly active compounds as well as the reference sulfonamides. The results are summarized in Table 2.

**TABLE 2:** Minimum Inhibitory Concentration of the most active compounds 1, 3, 4, 5, 6, 8, 11, 15, and 16.

Compounds	Minimum inhibitory concentration (µg/mL)	
	*E. coli*	*S. aureus*
1	512	512
2	1024	256
3	128	128
4	32	128
5	512	512
6	256	256
8	512	128
11	—	32
15	256	256
16	—	32
Sulfathiazole	>256	32–512
Sulfisoxazole	256	256

**FIGURE 2:**
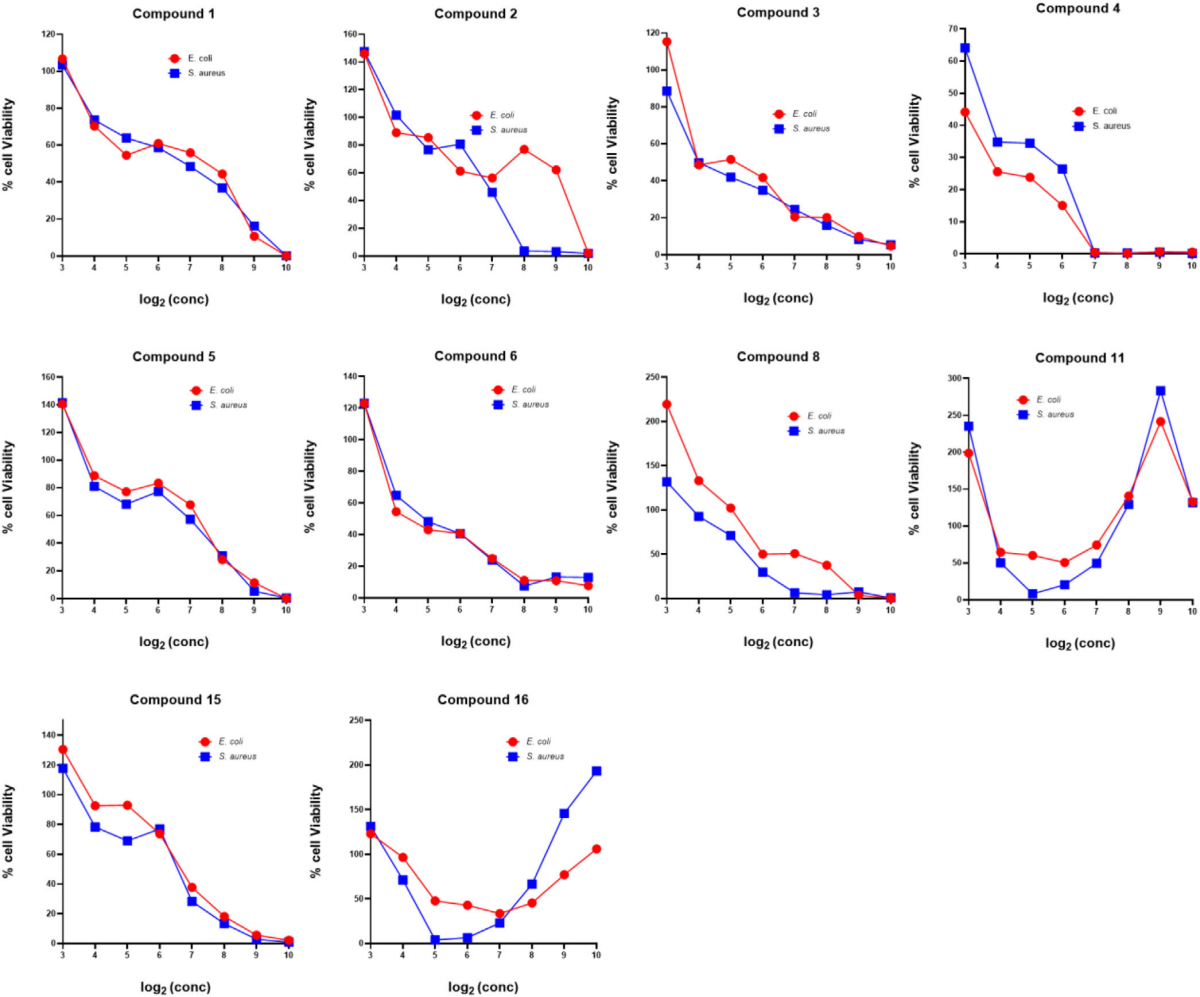
Dose–response curves of compounds 1–16 against *E. coli* CECT 101 (red) and *S. aureus* CECT 4013 (blue) showing % cell viability versus log_2_ (concentration, µg/mL). Concentrations are expressed on a log_2_ scale where values 3 to 10 correspond to 8, 16, 32, 64, 128, 256, 512, and 1024 µg/mL, respectively.

Consistent with the disk diffusion assay results, compound 4 was the only derivative exhibiting significant activity against *E. coli*, with an MIC_80_ of 32 µg/mL, compared with control sulfathiazole (MIC_80_ > 256 µg/mL). Dose–response analysis further revealed an MIC_50_ of 8 µg/mL for compound 4, further supporting its antibacterial potential (Figure 2).

Against *S. aureus* CECT 4013, compounds 11 and 16 exhibited the strongest antibacterial activity, each displaying MIC values of 32 µg/mL. Overall, the MIC values of the tested compounds ranged from 32 to 512 µg/mL, values broadly comparable to those of sulfathiazole.

Sulfonamide and sulfonyl motifs appear in over 150 FDA‐approved drugs and exhibit wide pharmacological diversity, including antibacterial, antiviral, antifungal, antitumor, antidiabetic, anti‐inflammatory, and CNS activities [24, 29, 30]. The growing interest in these applications has led to the synthesis of numerous sulfonyl‐containing analogues. However, interpreting their biological activity requires careful consideration of assay‐dependent factors.

Several compounds showed no activity in the disk diffusion, yet they were active in the broth dilution assay. The discrepancy observed between the disk diffusion and broth MIC results for certain compounds underscore how physicochemical properties can influence apparent activity. Disk diffusion relies on the compound diffusing through an agar matrix, a process that is sensitive to molecular size. For example, compounds 11 and 16 produced no zones on *S. aureus* plates yet demonstrated MIC values of 32 µg/mL in broth. These compounds have relatively high molecular weights (MW = 370–424 g/mol) and rigid aromatic structures, which may hinder their diffusion through agar. This phenomenon of poor agar diffusion for potent agents is well documented in antibiotic research [31]. The absence of an inhibition zone does not necessarily indicate a lack of intrinsic potency, particularly for less polar compounds, which diffuse more slowly in aqueous agar [31]. Conversely, compound 4, which is smaller and moderately polar, produced clear inhibition zones for *E. coli*, consistent with its favorable MIC value. However, formulating these compounds with solubilizing agents may improve their apparent activity in diffusion‐based assays.

The compounds exhibited a narrow‐spectrum antibacterial profile, with greater efficacy against *S. aureus* than against *E. coli*. Gram‐negative bacteria are generally less susceptible to sulfonamides because the outer membrane and efflux systems markedly restrict intracellular drug accumulation [32]. Compound 4 was the only compound active against *E. coli*, achieving an MIC of 32 µg/mL, representing an approximately eightfold improvement relative to the sulfonamide control. We attribute the activity of compound 4 against Gram‐negative bacteria partly to its favorable physicochemical profile: it is one of the smaller hybrids (MW = 366 g/mol) with relatively low polarity (TPSA = 83 Å^2^) and high predicted permeability, which likely facilitated its uptake across the *E. coli* outer membrane.

### 2.3 Minimum Bactericidal Concentration

Compounds 11 and 16 showed MBC values of 64 µg/mL, which are twofold higher than their respective MIC values, consistent with a bactericidal effect against *S. aureus* (Table 3). Compound 4 was the only compound for which both MIC (32 µg/mL) and MBC (256 µg/mL) values were determined, yielding a high MBC/MIC ratio of 8, indicative of a predominantly bacteriostatic mode of action. This is consistent with the well‐established bacteriostatic nature of sulfonamides [33]. These results encourage further investigation to elucidate the underlying mechanisms of action, thereby paving the way for the developing of more targeted antibacterial treatments.

**TABLE 3:** Minimum Bactericidal Concentration of the compounds 2, 4, 11, 15, and 16 against *S. aureus and E. coli*.

Compounds	Minimum bactericidal concentration (µg/mL)	
	*E. coli*	*S. aureus*
2	256	—
4	256	256
11	64	—
15	512	—
16	64	—

### 2.4 Pharmacokinetic Profile

Pharmacokinetic parameters of the tested compounds were predicted using both SwissADME and pkCSM web servers. These properties were interpreted based on standard reference ranges and compared with those of the control SMX.

#### 2.4.1 Prediction of Absorption

**FIGURE 3:**
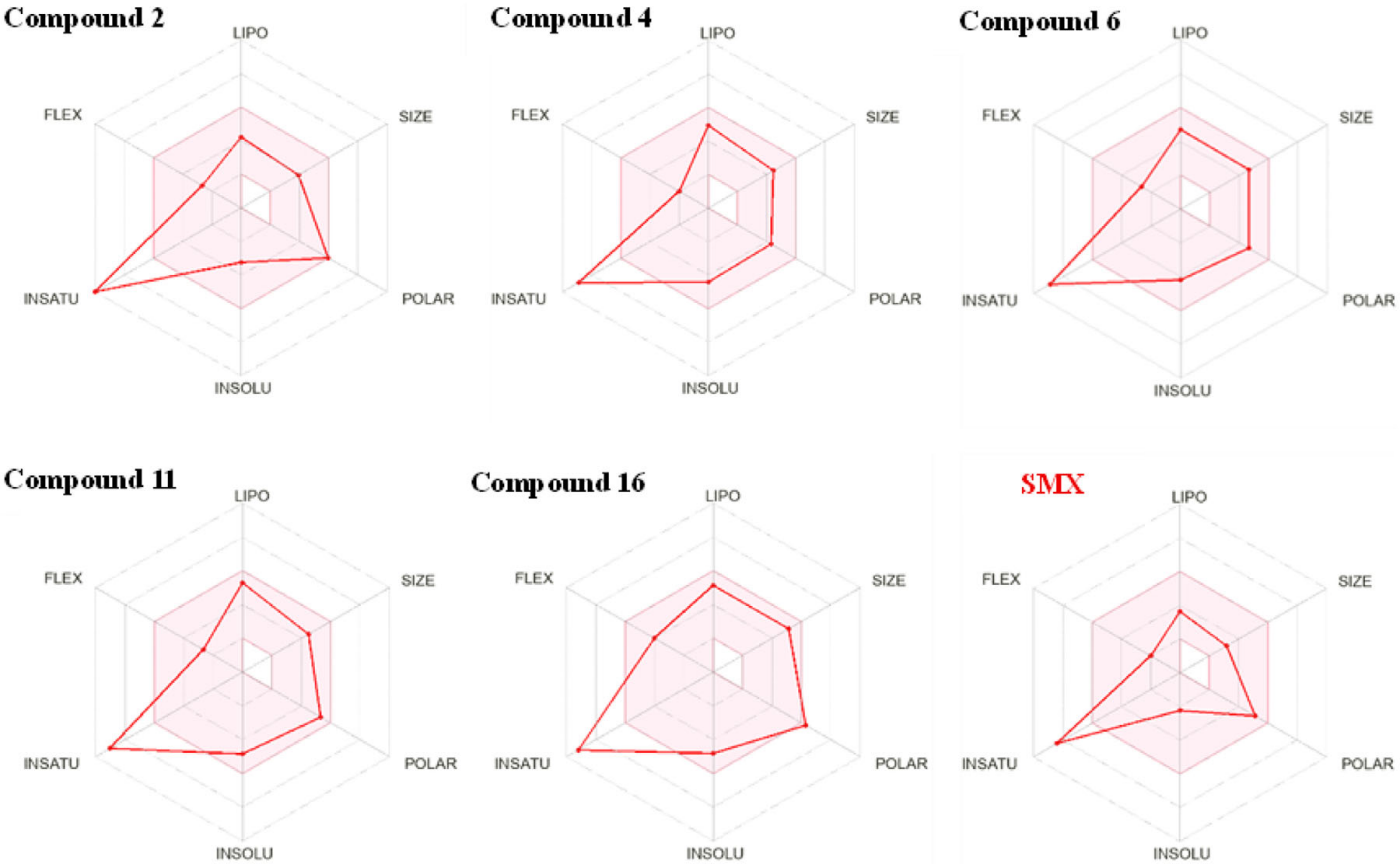
Bioavailability radar plots of compounds 2, 4, 6, 11, 16, and SMX.

Absorption determines the ability of a compound to reach systemic circulation following oral administration and directly governs its oral bioavailability. The SwissADME bioavailability radar plots indicate that all derivatives display a pronounced deviation in the unsaturation domain, reflecting their highly aromatic and rigid scaffolds, a characteristic feature of indazole‐based systems. Compounds 4, 6 and 11 exhibit the most balanced profiles, remaining largely within the optimal drug‐likeness space (the pink area) (Figure 3). In contrast, compound 2 and compound 16 show expanded polarity domains, consistent with their low absorption profiles. The absorption profiles of the compounds were evaluated using key parameters including lipophilicity (log P), water solubility (log S), gastrointestinal (GI) absorption, Caco‐2 permeability, and P‐glycoprotein (P‐gp) substrate status, with SMX included as a clinical reference. Compounds 2, 4, and 6 were the only derivates predicted to exhibit high GI absorption, similar to SMX. Caco‐2 permeability values varied across the series: compound 4 (0.964), compound 6 (0.926), and compound 11 (0.928) displayed higher permeability relative to SMX (0.128), while compound 15 (0.004), and compound 16 (0.057) exhibited lower permeability. Compounds with high Caco‐2 permeability will be more readily absorbed. P‐glycoprotein (P‐gp) is a member of the ATP‐binding cassette (ABC) transporter family involved in the efflux of drugs from cells. All compounds were predicted to be nonsubstrates of P‐gp, suggesting that they are unlikely to be actively transported out of cells and that their absorption is not mediated by P‐gp–dependent efflux mechanisms. Table 4 summarizes the absorption profiles of the most active compounds.

**TABLE 4:** Absorption profile of active indazole–sulfonamide derivatives compared with SMX.

Molecule ID	Absorption				
	logP	logS	GI absorption	Caco‐2 permeability	P‐gp substrate
2	1.12	−3.26	High	0.145	No
4	2.78	−4.41	High	0.964	No
6	2.48	−4.18	High	0.926	No
11	3.55	−4.84	Low	0.928	No
16	2.53	−4.80	Low	0.057	No
SMX	0.92	−2.25	High	0.128	No

#### 2.4.2 Prediction of Distribution

The distribution of drugs into tissues in vivo can be characterized by the steady‐state volume of distribution (VDss). Another key parameter is the fraction unbound (Fu), which represents the proportion of drug that remains free in plasma and unbound to plasma proteins, thereby remaining pharmacologically active. Compared with sulfamethoxazole (Fu = 0.366; VDss = 0.324), the indazole derivatives exhibited lower unbound fractions (Fu = 0.035–0.18) and markedly lower predicted VDss values (–0.003 to –0.792), indicating stronger plasma protein binding and very limited tissue distribution (Table 5).

**TABLE 5:** Distribution profile of active indazole–sulfonamide derivatives compared with SMX.

Molecule ID	Distribution		
	logP	logS	GI absorption
2	Fu	VDss	BBB
4	0.145	−0.246	No
6	0.077	−0.302	No
11	0.115	−0.003	No
16	0.035	−0.536	No
SMX	0.18	−0.279	No

#### 2.4.3 Prediction of Metabolism

The interaction of drugs with cytochromes P450 enzymes is essential for assessing drug elimination through metabolic biotransformation. All compounds were predicted to be substrates of CYP3A4 and CYP2D6, whereas SMX was predicted to be a nonsubstrate of both enzymes. Compound 11 was predicted to inhibit CYP3A4, while the remaining compounds showed no CYP3A4 inhibition. All compounds were predicted to inhibit CYP2C9, whereas SMX was predicted to show no CYP2C9 inhibition. Inhibition of major cytochrome P450 isoforms, including CYP1A2, CYP2C19, CYP2C9, CYP2D6, and CYP3A4, represents a primary mechanism underlying pharmacokinetic drug–drug interactions, which may result in reduced metabolic clearance and the subsequent accumulation of parent drugs or their metabolites, thereby increasing the risk of toxicity and other adverse effects (Table 6).

**TABLE 6:** Metabolism profile of active indazole–sulfonamide derivatives compared with SMX.

Molecule ID	Metabolism			
	CYP3A4 substrate	CYP2D6 substrate	CYP3A4 inhibitor	CYP2C9 inhibitior
2	Yes	Yes	No	Yes
4	Yes	Yes	No	Yes
6	Yes	Yes	No	Yes
11	Yes	Yes	Yes	Yes
16	Yes	Yes	No	Yes
SMX	No	No	No	No

#### 2.4.4 Prediction of Excretion

Excretion is the final stage of the ADME process and governs the elimination of a drug and its metabolites from the body, ultimately determining overall systemic exposure. The predicted total clearance values for the compounds ranged from 0.065 to 0.386 mL/min/kg, compared to 0.396 mL/min/kg for SMX. All compounds, including SMX, were predicted to be nonsubstrates of the renal Organic Cation Transporter 2 (OCT2) (Table 7).

**TABLE 7:** Excretion profile of active indazole–sulfonamide derivatives compared with SMX.

Molecule ID	Excretion	
	Total clearance	Renal OCT2
2	0.354	No
4	0.065	No
6	0.155	No
11	0.194	No
16	0.388	No
SMX	0.396	No

The indazole–sulfonamide hybrids demonstrated favorable drug‐likeness profiles, particularly regarding oral administration potential. Compounds 4, 6, and 11 showed high predicted GI absorption and Caco‐2 permeability, suggesting strong potential for systemic uptake. Their physicochemical properties, moderate lipophilicity, low polar surface area, and limited hydrogen bond donors, fall within the ideal range for orally administered drugs (Table 8). Most compounds also exhibited strong plasma protein binding and low predicted volumes of distribution, consistent with classical sulfonamides, which are known for extensive protein binding and wide distribution in various body fluids [33].

**TABLE 8:** Physicochemical properties of compounds 2, 4, 6, 11, and 16.

Mol ID	MW (g/mol)	Hydrogen bond acceptors	Hydrogen bond donors	Rotatable bonds	TPSA Å^2^
2	318.31	5	2	4	129.05
4	366.23	3	2	3	83.23
6	382.23	4	2	4	92.46
11	369.46	3	2	4	111.47
16	424.43	6	2	6	138.28
SMX	253.28	4	2	3	106.60

### 2.5 Prediction of Toxicity

The acute toxicity and safety profiles of the indazole–sulfonamide derivatives and the reference drug SMX were evaluated using the ProTox‐II web server (Table 9). The LD_50_ represents the median lethal dose required to induce mortality in 50% of the test population and serves as a standard indicator of acute toxicity and preliminary safety margin. All investigated compounds were classified into toxicity class 5 or 6, indicating low predicted acute oral toxicity. Regarding organ and genetic toxicity endpoints, ProTox‐II predictions classify compounds as active or inactive for each endpoint, where “active” denotes a predicted toxic liability and “inactive” denotes a low probability of inducing the corresponding toxic effect. Most compounds were predicted to be inactive for hepatotoxicity and cytotoxicity, with associated probabilities ranging from 0.51 to 0.84. However, the predicted moderate probabilities for carcinogenicity and mutagenicity observed for some derivatives suggest potential long‐term safety concerns that warrant further experimental validation. The predicted toxicity profiles indicate that these indazole–sulfonamide hybrids exhibit acceptable acute safety margins comparable to those of SMX, supporting their further investigation as antibacterial candidates.

**TABLE 9:** Toxicity prediction of active indazole–sulfonamide derivatives and SMX using ProTox‐II.

Compound ID	LD_50_ (mg/kg)	Toxicity class	Hepatotoxicity (Probability)	Carcinogenicity (Probability)	Mutagenicity (Probability)	Cytotoxicity (Probability)
2	2500	5	Inactive (0.54)	Active (0.64)	Active (0.58)	Inactive (0.80)
4	15800	6	Inactive (0.62)	Active (0.50)	Inactive (0.79)	Inactive (0.75)
6	2050	5	Inactive (0.51)	Inactive (0.54)	Inactive (0.69)	Inactive (0.74)
11	12500	6	Inactive (0.65)	Active (0.56)	Inactive (0.70)	Inactive (0.71)
16	2500	5	Active (0.51)	Active (0.58)	Active (0.70)	Inactive (0.84)
SMX	2300	5	Active (0.57)	Active (0.65)	Inactive (0.93)	Inactive (0.87)

### 2.6 Molecular Docking

Molecular docking is a widely applied in silico approach in structural biology and drug discovery that enables the prediction of ligand–receptor interactions and binding affinities at the atomic level. In this study, the indazole–sulfonamide hybrids that exhibited in vitro antibacterial activity were subjected to docking simulations against both wild‐type dihydropteroate synthase (WT‐DHPS) and the Sul1 mutant enzyme for in‐depth characterization of key interactions. This comparative analysis was performed to elucidate the impact of resistance‐associated mutations on ligand binding and to evaluate whether the designed compounds could overcome Sul1‐mediated resistance. SMX was additionally included as a reference drug to enable direct comparison with clinically used sulfonamides. Prior to screening, docking protocol validation was performed by redocking the co‐crystallized ligands of both target structures. For WT‐DHPS (PDB: 1AJ0), redocking of sulfanilamide yielded an RMSD of 0.371 Å relative to the crystallographic pose, and for the Sul1 variant (PDB: 7S2I), redocking of 6‐hydroxymethylpterin yielded an RMSD of 0.017 Å. Both values are well below the accepted validation threshold of 2.0 Å, confirming that the docking protocol reliably reproduces experimentally observed binding modes for both enzyme forms. The WT‐DHPS (PDB: 1AJ0), co‐crystallized with a sulfanilamide ligand (C_6_H_8_N_2_O_2_S), reveals that the binding pocket is formed by hydrogen–bond–forming residues Ser219, Arg220, and Arg63, along with aromatic residues Pro64, Phe190, Phe157, and Pro232 that contribute to hydrophobic and π‐stacking interactions. For the Sul1 mutant enzyme, the key binding residues identified in the crystal structure include Ile103, Asn101, Asp173, Lys212, Arg246, and Met125. The docking grid was defined to fully encompass the catalytic cavity, with grid box dimensions of 41.162 × 7.959 × 15.40 Å^3^, ensuring adequate conformational sampling of the active pocket. All compounds adopted stable conformations within the catalytic pocket and interacted with key residues of the active site through a combination of hydrogen bonding, π‐ π T‐shaped, π–cation, π–alkyl, π‐sulfur, and hydrophobic interactions.

**FIGURE 4:**
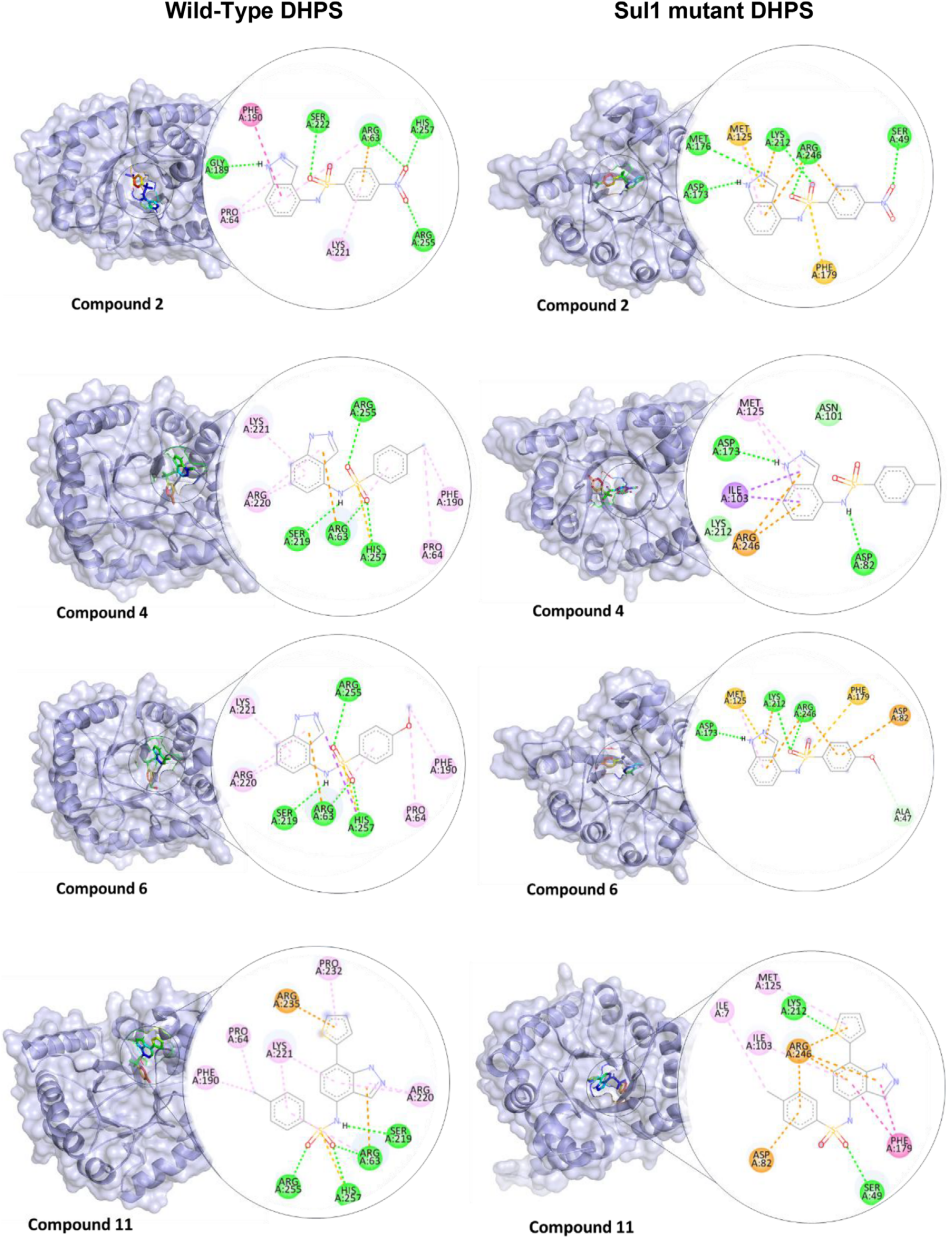
Molecular docking analysis of active indazole–sulfonamide derivatives within Wild‐Type and Sul1 mutant DHPS active sites.

Compound 4 demonstrated favorable binding affinity toward both WT‐DHPS (−7.53 kcal/mol) and the resistant Sul1 variant (−6.83 kcal/mol). Within the WT‐DHPS active site, hydrogen bond interactions were established with Arg63, Ser219, Arg255, and His257, highlighting the importance of these residues in ligand stabilization. Additional π–alkyl interactions were observed with Pro64, Phe190, Arg220, and Lys221. Compound 4 interacted with 5 of the 8 residues forming the active site of the wild type and with 5 of the 6 residues involved in the active site of the Sul1. For Sul1, hydrogen bonds were observed with Asn101, Asp173, and Lys212, while π–cation interactions involved Arg246 and π–alkyl interactions involved Ile103 and Met125. This interaction pattern suggests that compound 4 can effectively adapt to the mutated binding pocket. Notably, the indazole ring of compound 4 appears to engage in more interactions with the Sul1 mutant than with the wild‐type (Figure 4). This suggests that reconfiguration of the binding pocket in Sul1 favors additional contacts with the indazole moiety of compound 4, allowing it to retain or potentially optimize binding affinity despite the resistance‐associated structural shift. These findings position compound 4 as a promising candidate for resistance‐resilient DHPS inhibition.

Compound 11 exhibited the highest binding affinity toward WT‐DHPS with a binding energy of −7.79 kcal/mol, suggesting optimal shape and electronic complementarity with the binding cavity. Hydrogen bonds were formed with Arg255, His257, Arg63, and Ser219. A π–cation interaction with Arg235 and Pi–alkyl interactions with Arg220, Phe190, Pro64, Lys221, and Pro232 were also observed. Additionally, a π‐sulfur interaction with His257 contributed to enhanced ligand stabilization. The extended aromatic system of compound 11 enabled multiple noncovalent interactions, thereby explaining its superior binding affinity. In the Sul1 mutant DHPS, compound 11 maintained reasonable binding affinity (−6.54 kcal/mol) despite loss of key wild‐type contacts. The ligand formed hydrogen bonds with Ser49 and Lys212, as well as π–interactions with residues introduced or repositioned in the mutant site: π–π stacking with Phe179, Pi–cation interactions with Arg246 and Asp82, and π–alkyl interactions with Ile7, Ile103, and Met125.

Overall, all indazole–sulfonamide hybrids exhibited superior binding affinities relative to SMX against the wild‐type enzyme, indicating enhanced interaction profiles relative to the reference drug. Against the Sul1 mutant, compounds 4, 11, and 6 retained relatively high binding affinities, whereas compound 16 exhibited a notable reduction in binding affinity.

Several residues were recurrently involved in ligand binding across multiple compounds, including Arg63, Pro64, Arg255, His257, Ser219, Lys221, Phe190, and Arg220 in WT‐DHPS, and Asp173, Lys212, Arg246, Met125, and Asn101 in Sul1‐DHPS. The recurrent involvement of these residues underscores their critical role in ligand stabilization and catalytic function, highlighting them as key determinants for further structure‐based optimization of DHPS inhibitors.

All synthesized hybrids demonstrated more favorable binding energies compared with SMX, reflecting enhanced interaction capabilities within the DHPS active site. Although the binding energies remain within a moderate range, the designed compounds consistently surpass the binding characteristics of the commercially available sulfonamide reference. These findings indicate that structural modification through indazole conjugation enhances molecular recognition and binding stability within the DHPS active site. Table 10 presents a summary of key interactions and binding energies between the ligands and the target.

**TABLE 10:** Docking results of the active compounds in the active sites of Wild‐Type DHPS (WT) and mutant DHPS (Sul1).

Ligand	Target	Binding energy	Interactions					
			H‐bonds	H‐carbon	Pi‐pi T‐shaped	Pi‐cation	Pi‐alkyl	Pi‐sulfur
2	WT	−7.37	Arg63, Gly189, Ser222, Arg255, His257	—	Phe 190	—	Pro64, Lys221	—
	Sul1	−6.49	Ser49, Asp173, Met176, Lys212, Arg246	—	—	—	—	Met125, Phe179
4	WT	**−7.53**	Arg63, Ser219, Arg255, His257	—	—	—	Pro64, Phe190, Arg220, Lys221	—
	Sul1	**−6.83**	Asn101, Asp173, Lys212	—	—	Arg246	Ile103, Met125	—
6	WT	−7.3	Arg255, Ser219, Arg63, His257	—	—	—	Lys221, Arg220, Phe190, Pro64	—
	Sul1	−6.52	Asp173, Lys 212, Arg246	Ala47	—	Arg246, Lys212, Asp82	Met125	Phe179, Met125
11	WT	**−7.79**	Arg255, His257, Arg63, Ser219	—	—	Arg235	Arg220, Phe190, Pro64, Lys221, Pro232	His257
	Sul1	**−6.54**	Ser49, Lys212	—	Phe179	Arg246, Asp82	Ile7, Ile103, Met125	—
16	WT	−7.24	Thr62, Lys221	Ser222	Phe190	Arg235, Arg63	His257, Pro64	—
	Sul1	−5.04	Arg246, Asp82, Asn101, Ser208, Met176	Arg211	—	His248	Val223, Met125	—
SMX	WT	−5.84	Arg255, Thr62, Arg63, His257, Ser219			Arg63	Lys221, Pro232, Arg220	His257
	Sul1	−5.64	Asp173, Asn101, Arg246, Asp82			Arg246	Phe83, Ile103, Met125	Phe179

### 2.7 Structure‐Activity Relationship

Based on the docking simulations and antibacterial screening of the sixteen synthesized derivatives, several structure–activity relationship (SAR) trends can be inferred. In compound 4, halogen substitution (Br) enhances binding affinity, with the brominated aromatic ring contributing to hydrophobic and potential halogen‐bonding interactions, notably involving Arg63. This observation is consistent with previous studies, in which 2‐bromobenzene sulfonamides exhibited improved docking scores, and halogenation was consistently reported as a beneficial structural modification in DHPS inhibitors [34]. The antibacterial efficacy of sulfonamide derivatives is closely linked to their structural and physicochemical features. Electron‐withdrawing groups (e.g., halogens) on the aromatic ring generally increase antibacterial activity, while certain substitutions (e.g., methoxy groups) may reduce it [35, 36]. The Sul1 resistance determinant represents a clinically relevant resistance mechanism in which key structural alterations in DHPS reduce sulfonamide binding while preserving natural substrate affinity [10]. In the present study, compounds 4 and 11 retained moderate binding affinities to the Sul1 variant (–6.83 and –6.54 kcal/mol, respectively), suggesting that these molecules can accommodate the altered binding pocket. This suggests their potential to partially overcome sulfonamide resistance.

The *p*‐amino group on PABA and on sulfonamides such as SMX not only serves as a structural mimic but also provides a hydrogen‐bond donor in the DHPS active site. Structural studies have demonstrated that the hydroxyl group of the active‐site residue Thr51 in *S. aureus* DHPS forms hydrogen bonds with the amino group of PABA [6]. This interaction helps align PABA's amino group for nucleophilic attack during catalysis [11]. Since compound 4 lacks the *p*‐amino group, it is expected to lose this key hydrogen‐bond interaction with the enzyme. Future optimization could involve the introduction of a *p*‐amino (–NH_2_) group to restore this key interaction and potentially enhance antibacterial activity. Analogues of compound 4 incorporating this modification may constitute promising candidates against Gram‐negative bacteria.

Notably, two indazole‐sulfonamide compounds, compound 4 and 15, exhibit opposite species selectivity: compound 4 is active against Gram‐negative *E. coli* but inactive against Gram‐positive *S. aureus*, whereas compound 15 (the same scaffold bearing a nitro–NO_2_ substituent) is active against *S. aureus* but loses activity against *E. coli*. Both are presumed DHPS inhibitors, raising the question: How does the introduction of a nitro group shift activity from Gram‐negative to Gram‐positive bacteria? Several factors can contribute, including changes in electronic properties, differential membrane permeability, and efflux pump activity. The introduction of a nitro group onto the indazole‐sulfonamide scaffold constitutes a strongly electron‐withdrawing modification. Electron‐withdrawing substituents increase the acidity of sulfonamide nitrogen, and higher sulfonamide acidity correlates with greater antibacterial activity in classical sulfa drugs [37]. Multiple SAR studies confirm that in sulfonamide‐related scaffolds, the presence of a nitro substituent frequently confers or enhances anti‐*S. aureus* activity, whereas its removal or substitution markedly reduces activity [38, 39].

Another explanation for the inverse selectivity of compounds 4 and 15 is the fundamental difference in cell envelope architecture between Gram‐negative and Gram‐positive bacteria. *E. coli* is protected by an outer membrane that restricts the entry of many compounds, particularly large or highly polar molecules. Such bacteria rely on porin channels for uptake of hydrophilic nutrients and drugs; compounds above a certain size or with unfavorable polarity may not diffuse through porins effectively [32]. The nitro substituent increases the number of hydrogen bond acceptors and overall polarity; while this may improve binding to DHPS, it prevents passive diffusion across the lipid domains of the *E. coli* outer membrane. Indeed, it is well established that Gram‐negative bacteria are intrinsically less susceptible to nitro‐substituted compounds, largely because the outer membrane acts as a permeability barrier to their uptake [40].

The SAR trends described herein are consistent with those reported in the literature: electron‐withdrawing aryl sulfonyl groups increase sulfonamide acidity and reinforce DHPS hydrogen bonding, extended C7 aromatics boost π–π/cation–π contacts with residues like Phe190, Arg220, Lys221 (up to the limit imposed by DHPS pocket dimensions); and Gram‐negative activity requires a careful balance of molecular size and polarity, favoring smaller, amphipathic structures. Collectively, these principles support effective DHPS inhibition by indazole–sulfonamide hybrids.

## 3 Conclusions

The present study provides in vitro evidence and preliminary computational evidence suggesting that indazole–sulfonamide hybrids represent potential candidates for further development as promising DHPS‐targeting antibacterial agents. The most potent analogues combined a sulfonamide moiety with a heteroaryl indazole scaffold, yielding species‐specific inhibition: a small, bromo‐substituted hybrid (compound 4) exhibited enhanced activity against *E. coli*, while larger aromatic analogues (compound 11 and 16) were highly active and bactericidal against *S. aureus*. Structure–activity correlations indicate that introducing electron‐withdrawing, hydrophobic substituents on the phenyl sulfonamide ring (e.g., halogens) strengthens binding via hydrophobic and halogen bonding. Molecular docking revealed that these modifications yield numerous noncovalent contacts (hydrogen bonds, π–π and π–cation interactions) within the DHPS active site, improving potency over SMX. Notably, two hybrids (compound 4 and 11) maintained favorable predicted binding affinities against the Sul1‐resistant DHPS model, whereas a bulky derivative (compound 16) lost binding due to steric clash with the Sul1‐specific Phe insertion. These findings suggest design strategies for next‐generation sulfonamides: retain the PABA ‐mimicking sulfonamide core, append indazole‐ or phenyl‐rich substituents that engage the pterin subsite, and avoid excessive steric bulk near the pocket entrance. In silico ADME profiling indicated acceptable oral drug‐likeness for the lead compounds. Overall, the data provide a foundation for rational DHPS‐targeted drug design: by hybridizing the sulfonamide scaffold with privileged heterocycles, it is possible to enhance binding affinity, retain efficacy against resistant variants, and preserve a favorable pharmacokinetic profile. Future optimization may focus on the reintroduction of key functional groups such as a *para*‐amino (─NH_2_) substituent to fully mimic PABA interactions, and on experimental confirmation resistance‐escaping efficacy.

Several limitations of the present study should be acknowledged. First, the antibacterial evaluation was restricted to two reference strains, *E. coli* CECT 101 and *S. aureus* CECT 4013, which, while representative of Gram‐negative and Gram‐positive pathogens respectively and clinically relevant targets for sulfonamide‐based therapy, do not capture the full diversity of clinically encountered bacterial species and resistant isolates. Second, no direct enzymatic DHPS inhibition assay was performed, meaning that DHPS engagement remains a computationally supported hypothesis rather than an experimentally confirmed mechanism of action. Third, the evidence for resistance resilience is strictly computational, based on docking against the Sul1‐resistant DHPS crystal structure, and has not been validated against sul gene‐carrying resistant clinical isolates or through enzymatic inhibition of purified Sul1, Sul2, or Sul3 variants.

## 4 Experimental Section

### 4.1 Chemical Compounds

**FIGURE 5:**
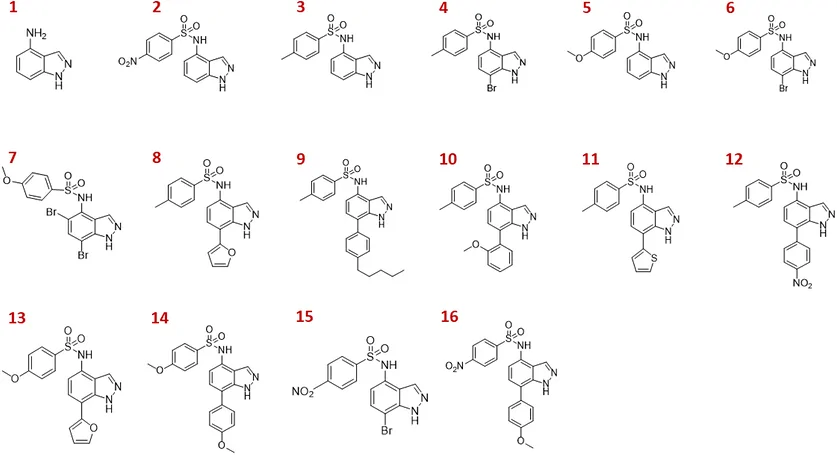
Substituted indazole sulfonamide derivatives tested for Antibacterial activity.

**FIGURE 6:**
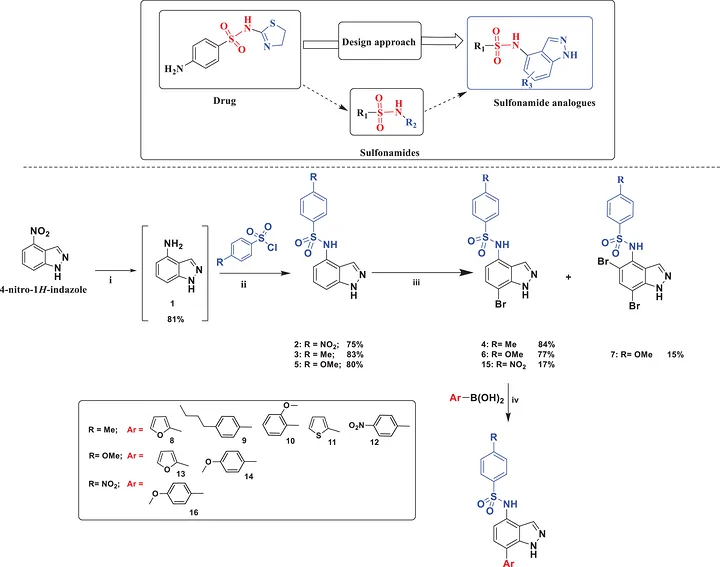
Reagents and reaction conditions for the synthesis of compounds 1–16. Reaction conditions: (i) Fe/NH_4_Cl (6 and 10 equiv., respectively), EtOH, rt, 3 h, 81%; (ii) ArSO_2_Cl (1 equiv.), pyridine, 24 h, 75%–83%; (iii) NBS (1.1 equiv.), DMF, reflux (80°C), 18 h; (iv) ArB(OH)_2_ (1 mmol), Pd(PPh_3_)_4_ (10 mol%), Cs_2_CO_3_ (1.3 mmol), dioxane/EtOH/H_2_O (3:1.5:0.5 mL), 140°C, 4 h. Adapted from Ref. [41].

A total of sixteen synthetic sulfonamide derivatives were kindly provided by the Medicinal Chemistry Laboratory, Euromed University of Fez (Figure 5). These compounds belong to a series of N‐(1H‐indazolyl) aryl sulfonamides, previously synthesized and fully characterized according to a published protocol. The chemical diversity of the series arises from different aryl sulfonyl groups (electron‐donating and electron‐withdrawing), different aryl or heteroaryl substituents at C7, which modulate the electronic and steric properties of the indazole core (Figure 6) [41]. Dimethyl sulfoxide (DMSO; analytical grade) was purchased from Sigma‐Aldrich (St. Louis, MO, USA) and used as the solvent. Commercial SMX antibiotic discs (50 µg) were obtained from Liofilchem (Roseto degli Abruzzi, Italy) and used as the positive reference antibacterial control.

### 4.2 Bacterial Strains

The bacterial strains *E. coli* CECT 101 (Gram‐negative) and *S. aureus* CECT 4013 (Gram‐positive) were obtained from the Spanish National Culture Collection (Paterna, Spain).

### 4.3 Preparation of Test Compounds

Each compound was initially dissolved in dimethyl sulfoxide (DMSO) to obtain a 5 mg/mL stock solution which was further diluted to 2.5 mg/mL and 1.25 mg/mL working solutions. These concentrations were selected to correspond to the amount of active substance loaded onto the positive control discs. A standard SMX disc contains 50 µg of the antibiotic. When 20 µL of a 2.5 mg/mL solution is applied onto a blank disc, the final loading equals 50 µg per disc. Similarly, a 5 mg/mL solution yields 100 µg/disc, and a 1.25 mg/mL solution provides 25 µg/disc to test a range of compound loadings for comparative purposes.

### 4.4 Agar Disc Diffusion Assay

Primary screening was performed using the agar disc diffusion method on Mueller–Hinton agar, following EUCAST guidelines [42, 43, 44]. Each compound was evaluated individually against *E. coli* CECT 101 and *S. aureus* CECT 4013. Each strain was cultivated separately in tryptic soy agar at 37°C overnight. Bacterial suspensions were prepared in sterile 0.85% saline and adjusted to a 0.5 McFarland standard (1–2 × 10^8^ CFU/mL), with turbidity verified spectrophotometrically at 625 nm (OD = 0.08–0.13) using an SP‐UV1100 spectrophotometer (Spectrum Instruments, Shanghai, China). Within 15 min of standardization, a sterile cotton swab was dipped into the suspension and uniformly streaked across the surface of the dried agar plates, which were then left to dry for 15 min with the lid closed. For each test, 20 µL of the compound solution was applied onto sterile blank discs and allowed to dry for 15 min under laminar airflow. Up to five discs were placed per plate. DMSO alone was used as a negative control to confirm that the solvent had no antibacterial effect, while SMX antibiotic discs served as a positive control. Plates were left at room temperature for 30 min to allow diffusion before incubation. After incubation, the inhibition zones were observed, and their diameters were measured in millimeters and compared with the standard antibiotic SMX, and interpreted as follows: ≤10 mm: no activity; 11 < D ≤ 15 mm: moderate activity and D > 15 mm: excellent activity.

### 4.5 Minimum Inhibitory Concentration (MIC)

The broth microdilution assay was used to evaluate the MIC of the synthesized compounds against *E. coli* CECT101 and *S. aureus* CECT 4013 following the protocol described in [45]. Test compounds were prepared at a stock concentration of 5 mg/mL using DMSO and subsequently diluted in Mueller–Hinton Broth to obtain the required test concentrations. Bacterial inoculum of *E. coli* CECT101 and *S. aureus* CECT4013 were adjusted to 0.5 McFarland (1–2 × 10^8^ CFU/mL) and further diluted to achieve a final inoculum of 5 × 10^5^ CFU/mL in each well. Serial twofold dilutions of each compound 1024, 512, 256, 128, 64, 32, 16, 8 µg/mL were prepared in 96‐well microtiter plates, and wells were inoculated with 50 µL of bacterial suspension. Plates were statically incubated at 37°C for 16–24 h. Optical density was measured at 600 nm using an Agilent BioTek 800 TS microplate reader (Agilent Technologies, Santa Clara, CA, USA). The MIC was determined as the lowest concentration that inhibits ≥80% of growth as compared to the growth control [46]. Sulfathiazole and Sulfisoxazole, functioning as a sulfonamide's antibiotics, were used in a comparative analysis. Each compound was evaluated in triplicate. The percentage of growth was calculated using the formula:

$$Viability=\frac{Absorbance\;of\;treated\;cells}{Absorbance\;of\;untreated\;cells}\times 100$$

### 4.6 Minimum Bactericidal Concentration (MBC)

Minimum bactericidal concentrations (MBCs) were determined from the MIC assay plates. After MIC reading, 100 µL aliquots from wells showing no visible growth were aseptically transferred onto Mueller–Hinton agar plates. The plates were incubated at 37°C for 18–24 h, and bacterial growth was assessed. The MBC was defined as the lowest concentration of each compound that resulted in ≥99.9% reduction in the initial inoculum, evidenced by the absence of colony formation on agar.

### 4.7 ADME

The compounds that show good in vitro activity were first evaluated for their suitability as drug candidates using standard drug‐likeness filters. Each compound was converted to its corresponding SMILES representation and analyzed through the SwissADME platform and pkCSM software, which provides predictions on physicochemical properties, pharmacokinetic behavior (ADME: absorption, distribution, metabolism, and excretion), medicinal chemistry parameters, and compliance with drug‐likeness rules [47, 48, 49]. Particular attention was given to Lipinski's rule of five, which proposes that orally active molecules should not exceed one violation among the following thresholds: molecular weight ≤500 Da, octanol/water partition coefficient (iLOGP) ≤ 5, no more than 10 hydrogen‐bond acceptors, no more than 5 hydrogen‐bond donors, and typically ≤10 rotatable bonds [47]. Additional parameters providing further insight into the pharmacokinetic profile of the compounds were also extracted. These parameters were used to identify derivatives with favorable oral bioavailability and adequate pharmacokinetic potential, ensuring that the most promising molecules could reach and interact with DHPS in sufficient concentrations to exert their biological effect.

### 4.8 Toxicity Prediction

In silico toxicity prediction was performed using the ProTox‐II web server (https://tox‐new.charite.de/protox_II, accessed 29 January 2026), which integrates molecular similarity, fragment‐based analysis, and machine‐learning models trained on experimentally validated toxicological data [50]. Compound structures were submitted in SMILES format, and acute oral toxicity was predicted as LD_50_ (mg/kg) with classification into toxicity classes I–VI (I = most toxic, VI = least toxic) according to the Globally Harmonized System (GHS). In addition, hepatotoxicity, carcinogenicity, mutagenicity, and cytotoxicity were evaluated, providing active/inactive predictions with associated probability scores to support comparative safety profiling and lead prioritization.

### 4.9 Molecular Docking

Molecular docking studies were conducted to investigate the binding interactions of the synthesized sulfonamide derivatives within the active site of DHPS. Docking was performed against both the wild‐type DHPS (PDB ID: 1AJ0) and a sulfonamide‐resistant sul1 variant (PDB ID: 7S2I), to compare binding modes and affinities in susceptible and resistance‐associated enzyme forms. Protein structures were retrieved from the Protein Data Bank and prepared for docking using AutoDock Tools by removal of co‐crystallized ligands and water molecules, followed by addition of polar hydrogens and assignment of Kollman charges [51, 52]. The two‐dimensional and three‐dimensional structures of the ligands were generated using Chem3D version 16.0 and energy‐minimized prior to docking. Docking simulations were carried out using AutoDock 4.0, employing the Lamarckian genetic algorithm to explore ligand conformational space [52]. The grid box was centered on the native substrate‐binding site of DHPS, encompassing the p‐aminobenzoic acid (PABA) and pterin‐binding regions. For each ligand, 40 independent docking runs were performed with default genetic algorithm parameters, allowing extensive conformational sampling. Binding poses were ranked according to predicted binding free energy, and the lowest‐energy conformations were selected for further analysis.

To validate the docking protocol, the co‐crystallized ligand of each target structure was extracted and redocked into its respective active site under identical docking parameters. The resulting poses were superimposed onto the original crystallographic conformations using PyMOL, and RMSD values were calculated to confirm reproducibility of the binding mode.

### 4.10 Statistical Analysis

The broth microdilution assay was performed in triplicate (n = 3), and results are expressed as mean ± standard deviation (SD). Cell viability (%) was calculated from OD_600_ measurements relative to the untreated growth control. The standard error of the mean (SEM) was calculated using Microsoft Excel (Microsoft Corp., Redmond, WA, USA). Dose–response curves were constructed using GraphPad Prism version 10.6.1 (GraphPad Software, San Diego, CA, USA).

## Author Contributions

**R. Boudza**: conceptualization, experiment design, data analysis, writing – original draft, biological experiments, data interpretation. **F. Elandaloussi** and **J. Segura‐Garcia**: molecular docking, data analysis, writing – review and editing. **R. Boudza** and **F. Elandaloussi**: biological experiments. **I. Moukadiri, S. Bounou** and **S. Maicas J. Segura‐Garcia**: supervision, resources, critical review, writing and editing. **S. El Kazzouli, N. El Brahmi**, and **K. Boujdi**: resources — critical review and editing. All authors approved the final manuscript.

## Conflicts of Interest

The authors declare no conflicts of interest.

## Use of Generative AI and AI‐Assisted Technologies in the Writing Process

During the preparation of this work the authors used ChatGPT in order to correct and improve the readability and language of the manuscript. After using this tool, the authors reviewed and edited the content as needed and take full responsibility for the content of the published article.

## Data Availability

The data that support the findings of this study are available from the corresponding author upon reasonable request.
